# *Bordetella parapertussis* Circumvents Neutrophil Extracellular Bactericidal Mechanisms

**DOI:** 10.1371/journal.pone.0169936

**Published:** 2017-01-17

**Authors:** Juan Gorgojo, Emilia Scharrig, Ricardo M. Gómez, Eric T. Harvill, Maria Eugenia Rodríguez

**Affiliations:** 1 CINDEFI (UNLP CONICET La Plata), Facultad de Ciencias Exactas, Universidad Nacional de La Plata, La Plata, Argentina; 2 Institute of Biotechnology and Molecular Biology, CCT-La Plata, CONICET-UNLP, La Plata, Argentina; 3 Center for Vaccines and Immunology, Department of Infectious Diseases, College of Veterinary Medicine, University of Georgia Athens, Georgia, United States of America; Hospital for Sick Children, CANADA

## Abstract

*B*. *parapertussis* is a whooping cough etiological agent with the ability to evade the immune response induced by pertussis vaccines. We previously demonstrated that in the absence of opsonic antibodies *B*. *parapertussis* hampers phagocytosis by neutrophils and macrophages and, when phagocytosed, blocks intracellular killing by interfering with phagolysosomal fusion. But neutrophils can kill and/or immobilize extracellular bacteria through non-phagocytic mechanisms such as degranulation and neutrophil extracellular traps (NETs). In this study we demonstrated that *B*. *parapertussis* also has the ability to circumvent these two neutrophil extracellular bactericidal activities. The lack of neutrophil degranulation was found dependent on the O antigen that targets the bacteria to cell lipid rafts, eventually avoiding the fusion of nascent phagosomes with specific and azurophilic granules. IgG opsonization overcame this inhibition of neutrophil degranulation. We further observed that *B*. *parapertussis* did not induce NETs release in resting neutrophils and inhibited NETs formation in response to phorbol myristate acetate (PMA) stimulation by a mechanism dependent on adenylate cyclase toxin (CyaA)-mediated inhibition of reactive oxygen species (ROS) generation. Thus, *B*. *parapertussis* modulates neutrophil bactericidal activity through two different mechanisms, one related to the lack of proper NETs-inducer stimuli and the other one related to an active inhibitory mechanism. Together with previous results these data suggest that *B*. *parapertussis* has the ability to subvert the main neutrophil bactericidal functions, inhibiting efficient clearance in non-immune hosts.

## Introduction

*Bordetella pertussis and Bordetella parapertussis* are human pathogens that cause whooping cough. Epidemiological studies revealed that *B*. *parapertussis* infections are globally more common than expected, affecting mainly vaccinated populations [1]. The incidence in vaccinated populations increased after introduction of acellular pertussis vaccines [2]. Current acellular pertussis vaccines, formulated with *B*. *pertussis* components, fail to protect against *B*. *parapertussis* infections [3–5]. Although cross-immunity could be expected between bacteria that share the majority of the vaccine antigens, the O antigen molecule on *B*. *parapertussis* surface blocks antibody access to the vaccine antigens common to both species [5]. Thus *B*. *parapertussis* largely avoids opsonophagocytosis and other activities mediated by vaccine-induced antibodies [5–7], surviving and growing primarily extracellularly within the respiratory tract. Avoiding antibody-mediated effects does not prevent exposure to the many other anti-microbial activities unleashed upon invading bacteria. Neutrophils are rapidly recruited to the site of bacterial infection. These cells are equipped with extracellular bactericidal mechanisms, such as degranulation and NETs [8, 9]. Degranulation occurs when specific membrane receptors are activated and neutrophil cytoplasmic granules translocate to the plasma membrane [10], or when during bacterial phagocytosis, nascent unclosed phagosomes fuse with granules releasing their content into the extracellular medium [11, 12]. On the other hand, NETs have been observed *in vivo* during infection with bacteria [9, 13, 14], fungi [15, 16], protozoa [17, 18], and viruses [19–21]. The microbicidal activity of NETs is mediated by the same components released via degranulation, including neutrophil elastase (NE), myeloperoxidase, cathepsin G, proteinase 3, bacterial permeability-increasing protein, lactoferrin, gelatinase, the cathelicidin LL-37, and tryptase with the addition of nuclear antimicrobial factors as DNA and histones [22, 23]. Therefore, the key difference between NETs release and degranulation is that the NETs, through their network structure, act as a physical container of microbes that reduces their dissemination, allowing the microbicidal factors to exert their action more efficiently, and limiting the damage to the surrounding tissues by confining antimicrobial agents to a restricted area [9, 24]. NETs released may be induced by several stimuli including bacterial components, proinflammatory cytokines or PMA [9, 23]. Most of the NETs inducer stimuli initiate NETs release through ROS generated by the activation of the NADPH oxidase (NOX) system. ROS lead to the translocation of NE from azhurophilic granules to the nucleus where it initiates several chromatin structure rearrangements leading to chromatin decondensation. Next, the nuclear envelope is dismantled and chromatin is mixed with bactericidal compounds of cytoplasmic, granular, and nuclear origin. Finally, the cell membrane is broken and NETs are released into the extracellular medium trapping and/or killing bacteria [25, 26]. Importantly, NETs were found released in the respiratory tract in response to several pathogen infections [16, 19, 27–29], although their role in whooping cough is still uncertain.

In the present study, we investigated neutrophils extracellular antimicrobial functions against *B*. *parapertussis* and explored how this pathogen is able to resist these functions. Our results indicate that *B*. *parapertussis* is able to evade neutrophil extracellular killing mechanisms if specific antibodies are not present at the site of interaction.

## Materials and Methods

### Bacterial strains and growth

The *B*. *parapertussis* strain CN2591 and the isogenic *B*. *parapertussis* mutant strain lacking the O antigen, CN2591Δwbm [30, 31], were used in this study. Bacteria were stored at -70°C and recovered by growth on Bordet-Gengou agar (BGA) (BD Difco) plates supplemented with 15% defibrinated sheep blood (Laboratorio Argentino, Caseros, Argentina) (bBGA) for 24 h at 36°C. Animal handling and all procedures were in compliance with the Argentinean animal protection Law 14346. Virulent (Bvg^+^) bacteria were subsequently seeded on Stainer-Scholte (SS) liquid medium, cultured for 20 h at 36°C, and used in the experiments. Avirulent (Bvg^-^) *B*. *parapertussis* was obtained by growing the bacteria on bBGA plates supplemented with magnesium sulfate at a final concentration of 50 mM for 24 h at 36°C, subsequently seeded on SS liquid medium containing 50 mM magnesium sulfate, cultured for 20 h at 36°C, and used in the experiments. Transition from Bvg^+^ to Bvg^-^ phase was confirmed by the loss of expression of the CyaA and the filamentous hemagglutinin by western blot analysis of whole-cell lysates as previously described [32] (data not shown). Inactivation of *B*. *parapertussis* was obtained by treating bacterial cells with 4% (w/v) paraformaldehyde for 1 h, washed once with phosphate-buffered saline (PBS), incubated for 10 min at room temperature with PBS containing 50 mM NH_4_Cl, washed, and suspended in the appropriate medium. *Pseudomona aeruginosa* strain PA01 was grown for 12 h at 37°C in Luria–Bertani medium (Gibco, Rockville, MD).

### Cells

Human neutrophils were isolated from heparinized venous blood using Ficoll-Histopaque (Sigma, St Louis, MO) gradient centrifugation. Neutrophils were harvested and the remaining erythrocytes were removed by hypotonic lysis. Cell viability was 99% as determined by Trypan blue exclusion. Prior to functional assays, neutrophils were washed twice with Dulbecco’s modified Eagle medium (DMEM) (Gibco) supplemented with 10% heat-inactivated fetal calf serum (FCS) (Gibco), suspended, and used immediately (the presence of heat-stable nucleases in FCS was discarded as in [33]). All experiments were carried out with freshly isolated neutrophils lacking FcγRI expression, as monitored by FACS analysis using a fluorescence-activated cell sorter FACScalibur flow cytometer with anti-hFcγRI mAb 22 (mIgG1) [34]. Data were processed using the CellQuest software (BD Biosciences, San Jose, CA). All procedures involving human samples were in accordance with the ethical standards of the 1964 Helsinki declaration and its later amendments, and approved by the Institutional Review Board. Peripheral blood was collected from healthy donors. All individuals provided written informed consent for the collection of samples and subsequent analysis.

### Antibodies

The following antibodies were used: mouse anti-human LAMP-1 monoclonal antibodies (Pharmingen, San Diego, CA), rabbit anti-human flotillin-1 (Santa Cruz Biotechnology, Santa Cruz, CA), rabbit anti-human NE polyclonal antibodies (Calbiochem-Merk Millipore; Darmstad, Germany), mouse anti-hFcγRI (CD64) MAb 22 (mIgG1) (Medarex, Annandale, NJ). Cy3-conjugated goat F(ab´)_2_ fragments of anti-rabbit IgG and fluorescein isothiocyanate (FITC)-conjugated goat F(ab´)_2_ fragments of anti-rabbit IgG (both from Jackson ImmunoResearch, West Grove, PA), Cy3-conjugated goat F(ab´)_2_ fragments of anti-mouse IgG, phycoerythrin (PE)-conjugated goat F(ab´)_2_ fragments of anti-mouse IgG (both from Molecular Probes, Eugene, OR), and FITC-conjugated goat F(ab´)_2_ fragments of anti-mouse IgG (from Southern Biotechnology, Birmingham, AL). Immunoglobulin G (IgG) fractions from pooled sera of whooping cough patients with high titers of anti-*B*. *parapertussis* antibodies, as measured by enzyme-linked immunosorbent assay [35], were isolated as described previously [36]. Polyclonal rabbit anti-*B*. *parapertussis* antiserum and polyclonal mouse anti-*B*. *parapertussis* antiserum were obtained as described elsewhere [37].

### Respiratory burst determination

ROS production by neutrophils was determined as previously described [7], with minor modifications. Briefly, tubes containing 2x10^7^ bacteria were transferred to a Luminometer (Luminoskan TL Plus, Thermo LabSystems, Finland), in which chemoluminescence response of 2x10^5^ neutrophils was measure every minute for 120 min at 37°C after injection of 120 μM luminol (Sigma). Neutrophils incubated with PMA were used as positive control. Neutrophils incubated with diphenyleneiodonium (DPI), a NOX inhibitor, 30 min before PMA treatment were used as a control of inhibition of PMA-induced ROS.

### Neutrophil degranulation studies

Exocytosis of the lysosomal enzyme β-glucuronidase was determined as previously described [38], with minor modifications. Briefly, 2x10^5^ neutrophils were incubated with bacteria at a multiplicity of infection (MOI) of 300 for 10 min at 37°C and centrifuged at 300 x g. Supernatants were further centrifuged at 10,000 x g for 10 min to eliminate bacteria. β-glucuronidase activity was measured at 405 nm in cell lysed with 0.2% (v/v) Triton X-100 (Sigma), and in the cell-free supernatants. Controls were performed by determination of β-glucuronidase activity from non-infected 2x10^5^ neutrophils lysed by Triton X-100 treatment, assumed as a 100% of release. Cell-free supernatant of uninfected neutrophils was used as a negative control.

Confocal microscopy studies were performed as previously described [7], with minor modifications. Briefly, neutrophils were incubated with *B*. *parapertussis* at a MOI of 300 at 37°C. Ten minutes post infection neutrophils were incubated with or without 200 nM LysoTracker DND-99 (Molecular Probes) (5 min at 37°C) prior to fixation with 4% (w/v) paraformaldehyde. Samples incubated with LysoTracker stain were then washed twice with PBS and further incubated 10 min at room temperature with PBS containing 50 mM NH_4_Cl. After two washing steps, the cells were incubated for 30 min at 25°C with PBS containing 0.1% (w/v) saponin (Sigma) and 0.2% (w/v) bovine serum albumin (BSA) (Sigma), followed by incubation with polyclonal rabbit anti-*B*. *parapertussis* antiserum (30 min at 25°C) in the presence of 0.1% (w/v) saponin and 0.2% (w/v) BSA. After three washing steps, neutrophils were incubated with FITC-conjugated goat F(ab´)_2_ fragments of anti-rabbit immunoglobulin (30 min at 25°C). Samples of infected cells that were not incubated with LysoTracker stain were washed twice with PBS and incubated for 10 min at room temperature with PBS containing 50 mM NH_4_Cl. After two washing steps, the cells were further incubated for 30 min at 25°C with PBS containing 0.1% (w/v) saponin and 0.2% (w/v) BSA. Next, the cells were incubated for 30 min at 25°C with either mouse anti-human LAMP-1 monoclonal antibodies plus polyclonal rabbit anti-*B*. *parapertussis* antiserum or rabbit anti-human flotillin-1 plus polyclonal mouse anti-*B*. *parapertussis* antiserum antibodies (30 min at 25°C) in the presence of 0.1% (w/v) saponin and 0.2% (w/v) BSA. After three washing steps, the cells were incubated with either Cy3-conjugated F(ab´)_2_ fragments of goat anti-mouse antibodies plus FITC-conjugated goat F(ab´)_2_ fragments of anti-rabbit immunoglobulin or Cy3-conjugated F(ab´)_2_ fragments of goat anti-rabbit antibodies plus FITC-conjugated goat F(ab´)_2_ fragments of anti-mouse immunoglobulin for another 30 min at 25°C. To avoid cytophilic binding of antibodies to the FcR, all incubations were done in the presence of 25% (v/v) heat-inactivated human serum. Additionally, isotype controls were run in parallel. Microscopic analyses were performed using a confocal laser scanning microscope (model TCS SP5; Leica, Mannheim, Germany). The percentage of phagosomes containing the bacterium that colocalized with a given marker was calculated from the number of total intracellular bacteria by analyzing at least 50 phagosomes per donor.

In control studies neutrophils were incubated with 10 mg/ml of β-methyl cyclodextrin (Sigma) (15 min at 37°C) in serum-free DMEM plus bovine serum albumin (BSA) (0.2%) and lovastatin (5 μg/ml) (Sigma) (DMEM-BSA-L), as previously described [7]. Cells were then washed, suspended in DMEM-BSA-L, and used immediately in infection studies. No decrease in neutrophils viability was detected after treatment.

### NETs formation studies

NETs formation studies were performed as previously described [9], with minor modifications. Briefly, 2x10^5^ neutrophils were seeded in 24 well-plates containing 12 mm coverslip and incubated with bacteria at different MOI (10, 50 or 100), centrifuged for 5 min at 300 × g, and further incubated at 37°C for different time periods. Neutrophils incubated with or without PMA (Sigma) (20 nM) were used as positive and negative control, respectively. In some experiments, neutrophils were incubated with either bacteria or conditioned medium (CM) (obtained as described below) for 30 min at 37°C before incubation with PMA (20 nM). Neutrophils incubated with DPI 30 min before PMA treatment were used as a control of inhibition of PMA-induced NETs release. The NETs were visualized by immunofluorescence microscopy as described below.

Fluorescence microscopy was used to visualize NETting cells. Immunostaining of cells was performed as described before [7], with minor modifications. At different time points samples were fixed with 4% (w/v) paraformaldehyde, washed twice with PBS, and incubated for 10 min at room temperature with PBS containing 50 mM NH_4_Cl. After two washing steps, the cells were incubated for 30 min at 25°C with PBS containing 0.1% (w/v) saponin and 0.2% (w/v) BSA. Next, the cells were incubated with rabbit anti-human NE polyclonal antibodies (30 min at 25°C) in the presence of 0.1% (w/v) saponin and 0.2% (w/v) BSA. After two washing steps, neutrophils were incubated with FITC-conjugated goat F(ab´)_2_ fragments of anti-rabbit immunoglobulin in the presence of 0.1% (w/v) saponin and 0.2% BSA (w/v) for another 30 min at 25°C, and washed twice. Finally, the samples were incubated with propidium iodide (Sigma) for DNA stain at a concentration of 2 mg/ml in PBS for 5 min. After washing with distillated water, coverslips were mounted on microscope slides and analyzed by fluorescence microscopy using a DMLB microscope coupled to a DC100 camera (Leica Microscopy Systems Ltd., Heerbrugg, Switzerland).

In order to evaluate if NETs can immobilize *B*. *parapertussis*, 2x10^5^ neutrophils were seeded in 24 well-plates containing 12 mm coverslip and then stimulated with PMA 100 nM for 1 h to induce NETs release. Neutrophils were incubated with bacteria for 3 h, washed and fixed with 4% (w/v) paraformaldehyde. NE and DNA were stained as above described. After washing with distillated water, coverslips were mounted on microscope slides and analyzed by confocal laser scanning microscope (model TCS SP5; Leica).

### NETosis quantification

Quantification of neutrophils that underwent NETosis was performed using the image processing software, ImageJ (National Institutes of Health, Bethesda, Maryland, USA, http://imagej.nih.gov/ij/), as previously described [39] with minor modifications. Briefly, fluorescence microscopy images captured with a 40X objective were used to measure individual nuclear area of neutrophils stained with propidium iodide by adjusting a threshold above background. The average value of nuclear area of resting neutrophils was chosen as a cutoff value. Cell nucleus with areas larger than this cutoff value were considered NETting cells. This correlation was confirmed by checking whether randomly chosen cells which nuclear area exceeding the cutoff value showed chromatin colocalizing with the major NETs-associated granular protein NE. Results were obtained from three independent experiments run in duplicate using neutrophils from three different donors. At least 500 cells from each condition were examined.

### Conditioned medium

CM was obtained as follows. Bacteria were grown in SS medium, washed, and further incubated in DMEM plus 10% FCS at a concentration of 2×10^8^ bacteria/ml for 2 h at 37°C. Next, the medium was filtered with a 0.22 μm membrane filter to remove the bacteria and further used in experiments. In selected experiments CM was treated with a monoclonal antibody against both activities of CyaA (2A12), a monoclonal antibody that prevents translocation of the enzyme catalytic domain into the cell (5D1), or a monoclonal antibody against the hemolytic activity (6E1) of the CyaA [40], all kindly supplied by Erik L. Hewlett, Division of Infectious Diseases and International Health, Department of Medicine, University of Virginia, Charlottesville, Virginia, USA. In other experiments CM was treated with proteinase K (PK) 100 μg/mL for 60 min at 37°C before used.

### DNA degradation studies

DNase activity of *B*. *parapertussis* was determined as previously described [41], with minor modifications. Briefly, purified neutrophil DNA (300 ng) was incubated at 37°C for 60 min with PBS (negative control), 500 mU/ml of Micrococcal nuclease I (MNase I) (Roche Diagnostics; Mannheim, Germany) (positive control), *B*. *parapertussis* (6x10^9^ colony forming units (CFU)/ml), or bacterial-free culture supernatant (obtained by filtration of a 20 h bacterial culture in SS medium). Next, samples were mixed with sample buffer and loaded onto a 1% agarose gel and run in TBE buffer with 1% ethidium bromide at 100 volts.

### Bacterial killing by NETs

The ability of NETs to kill *B*. *parapertussis* was evaluated as previously described [42] with minor modifications. Briefly, 2×10^5^ neutrophils were seeded in 24 well-plates and then incubated with 100 nM PMA for 1 h at 37°C, to induce NETs. Next, neutrophils were treated or not with 100 μg/ml Cytochalasin D (CytD) (Sigma) to inhibit phagocytosis or with 500 mU/ml MNase I, to breakdown NETs, for 30 min. In control experiments a combination of CytD and MNase I was used. Neutrophil were then incubated with bacteria (MOI:100) for 3 h and further treated with MNase I for 20 min to release the bacteria eventually immobilized by NETs. Neutrophils were further subjected to hypotonic lysis by using 0.1% saponin in sterile deionized water and serial dilutions of lysates were rapidly plated onto bBGA to enumerate the CFU. To confirm that NETs can kill *B*. *parapertussis* a microscopic analysis using a LIVE/DEAD Cell Viability Assays Kit (Molecular Probes) was performed according to the manufacturer’s instructions.

### Statistical analysis

Student’s t test (95% confidence level) or analysis of variance (ANOVA) was used for statistical data evaluation. The significance of the differences between the mean values of the data evaluated by ANOVA was determined with the least-significant-difference (LSD) test at a 95% confidence level. Results are shown as means and standard deviations (SD).

## Results

### *B*. *parapertussis* inhibits neutrophil degranulation

**Fig 1:**
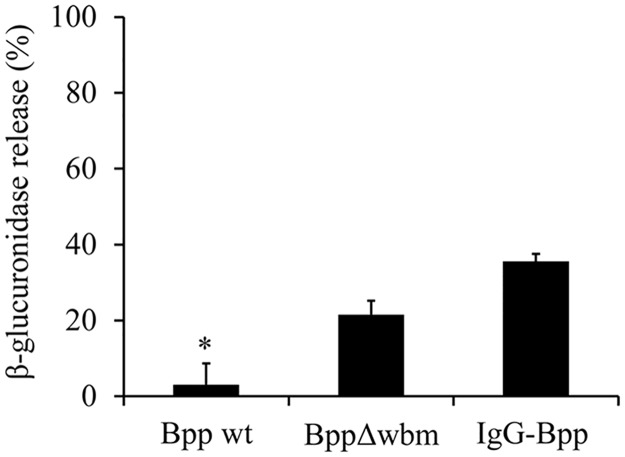
Non-opsonized *B parapertussis* fails to trigger neutrophil degranulation. Neutrophils were incubated with non-opsonized *B*. *parapertussis* (Bpp), non-opsonized *B*. *parapertussis* Δwbm (BppΔwbm), or IgG-opsonized *B*. *parapertussis* (IgG-Bpp) at a MOI of 300. The release of β-glucuronidase 10 min post infection was expressed as a percentage of the total β-glucuronidase cell content. The data represent the means ± SD of three independent experiments. The β-glucuronidase activity in the cell free supernatant of neutrophils infected with non-opsonized *B*. *parapertussis* was significantly different from the β-glucuronidase activity in the cell free supernatant of neutrophils infected with opsonized *B*. *parapertussis* or the O antigen deficient *B*. *parapertussis* (*P <0.05).

We previously demonstrated that in the absence of opsonic antibodies, *B*. *parapertussis* avoids neutrophil intracellular killing by inhibiting phagolysosomal fusion through an O antigen dependent mechanism [7]. In this study, we investigated whether these interactions modulate neutrophil degranulation. To this end, human neutrophils were incubated with non-opsonized *B*. *parapertussis*, non-opsonized O antigen deficient *B*. *parapertussis* or IgG-opsonized *B*. *parapertussis* at a MOI of 300. Ten minutes after exposure the azurophil granule enzymeβ-glucuronidase activity in the cell-free supernatants was measured. Supernatant of uninfected neutrophils was used as negative control. Total β-glucuronidase activity of uninfected neutrophils was determined and set as 100%. As can be seen in Fig 1, non-opsonized *B*. *parapertussis* failed to trigger significant neutrophil degranulation. In contrast, the *B*. *parapertussis* mutant lacking the expression of the O antigen induced strong neutrophil degranulation, suggesting that O antigen blocks degranulation. The ability of *B*. *parapertussis* to impair phagosome-lysosome maturation was previously found abrogated by IgG opsonization of the bacteria [7]. Accordingly, Fig 1 (S1 Data) shows that IgG-opsonized *B*. *parapertussis* also induced neutrophil degranulation.

**Fig 2:**
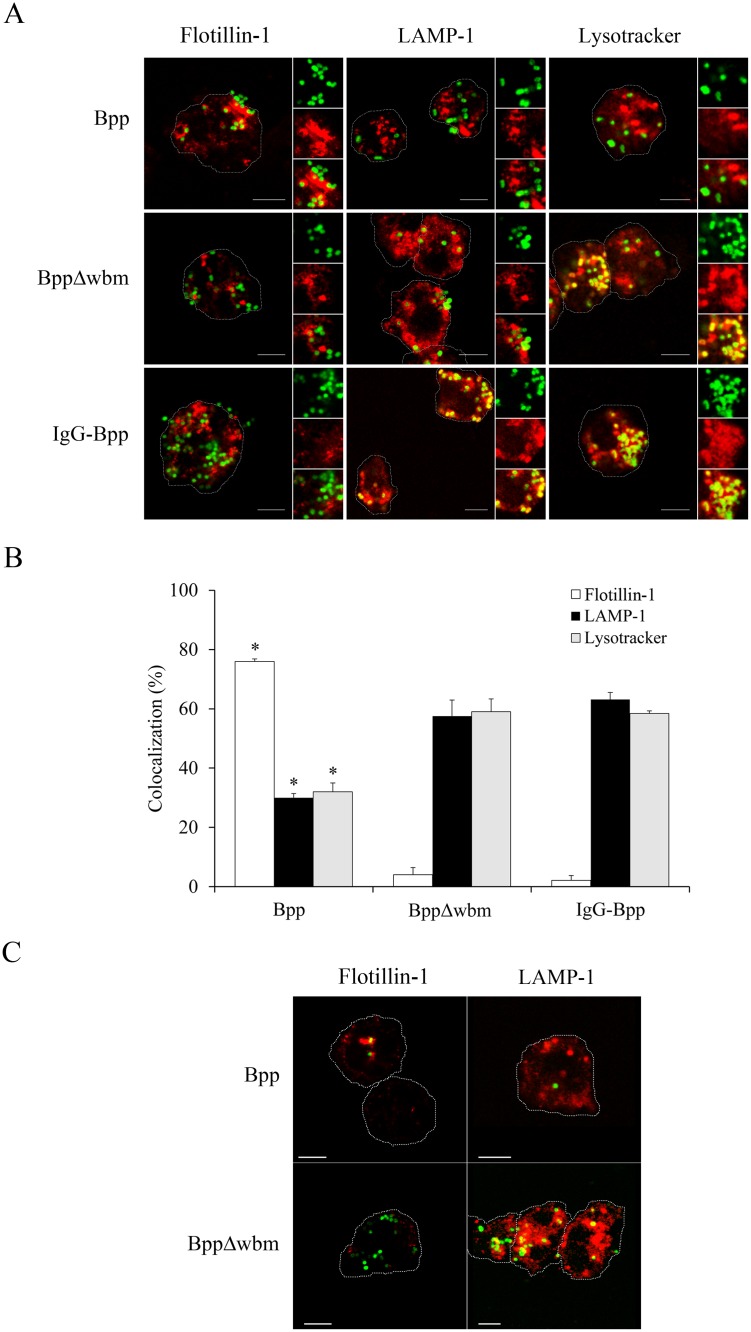
*B*. *parapertussis* entry through lipid rafts prevents fusion of nascent phagosomes with specific and azhurophil granules. Neutrophils were incubated with non-opsonized *B*. *parapertussis* (Bpp), non-opsonized *B*. *parapertussis* Δwbm (BppΔwbm), or IgG-opsonized *B*. *parapertussis* (IgG-Bpp) at a MOI of 300 for 10 min at 37°C. After washing, samples were incubated with or without Lysotracker (azhurophil granule marker), fixed and permeabilized prior to incubation with antibodies against *B*. *parapertussis* to FITC stained the bacteria. Samples that were not incubated with LysoTracker were fixed and permeabilized prior to incubation with antibodies against either flotillin-1 (red) (lipid raft marker) or LAMP-1 (red) (specific granule marker). *B*. *parapertussis* was FITC stained by using antibodies against bacteria. Finally samples were analyzed by confocal microscopy. (A) Colocalization of bacteria with lipid rafts is reflected by green-fluorescent *B*. *parapertussis* surrounded by red labeled flotillin-1. Bacterial interaction with either specific or azhurophilic granules is reflected by green-fluorescent *B*. *parapertussis* surrounded by red labeled LAMP-1 or Lysotracker positive compartments, respectively. Representative panels of one of three independent experiments are shown. Scale bar: 5 μm. (B) The bars indicate the respective percentages of bacteria colocalizing with a given marker. The data represent the means ± SD of three independent experiments. At least 50 phagosomes were counted per sample. The percentage of colocalization of non-opsonized *B*. *parapertussis* with a given marker was significantly different from the percentage of colocalization with the respective marker in neutrophils infected with either the non-opsonized *B*. *parapertussis* Δwbm or the IgG-opsonized *B*. *parapertussis* (*P <0.05). (C) Neutrophils were treated with a cholesterol-depleting drug (β-methyl cyclodextrin, 10 mg/ml) prior to incubation with Bpp or BppΔwbm (both at an MOI of 300) for 10 min at 37°C. After washing, cells was fixed and permeabilized prior to incubation with antibodies against *B*. *parapertussis* to FITC stain the bacteria and antibodies against either flotillin-1 (red) or LAMP-1 (red). Representative panels of one of three independent experiments are shown. Scale bar: 5 μm.

We previously found that the interaction of *B*. *parapertussis* with lipid rafts through the O antigen was involved in inhibition of phagosome-lysosome fusion [7]. We examined this interaction in relation to neutrophil degranulation by confocal microscopy using flotillin-1 as lipid raft marker and Lysotracker and LAMP-1 as markers of azurophil [43] and specific granules [44], respectively. Fig 2 (S2 Data) shows that as early as 10 min post infection, a high number of wild type bacteria were mainly colocalizing with flotillin-1, and only a small percentage of bacteria were found colocalizing with LAMP-1 or Lysotracker. On the other hand, at the same time point post infection, neither the O antigen deficient mutant nor the IgG-opsonized bacteria were found colocalizing with flotillin-1, whereas they did colocalize with the LAMP-1 or Lysotracker markers (Fig 2). In control experiments we further confirmed that, as previously found [7], the treatment of neutrophils with β-methyl cyclodextrin, a compound that disrupts cholesterol-rich domains by extracting cholesterol, did not affect the attachment of the O antigen deficient mutant but did induce a significant decrease in the number of attached wild type *B*. *parapertussis*. Neutrophils treatment with β-methyl cyclodextrin, on the other hand, did not affect the colocalization of LAMP-1 positive compartments with the O antigen deficient mutant strain. Neither was affected the level of colocalization of the wild type strain of *B*. *parapertussis* with LAMP-1 positive compartments in β-methyl cyclodextrin treated neutrophils, which is in agreement with the finding that the few bacteria found in these neutrophils were colocalizing with the residual flotillin-1 positive spots (Fig 2C). Accordingly, β-glucuronidase release was not modified by treatment of neutrophils with β-methyl cyclodextrin prior to incubation with either the wild type strain or the O antigen deficient mutant (data not show). All together, these results suggest that *B*. *parapertussis* inhibits neutrophil degranulation via an O antigen- lipid raft- dependent mechanism.

### *B*. *parapertussis* does not induce NETs in resting neutrophils

**Fig 3:**
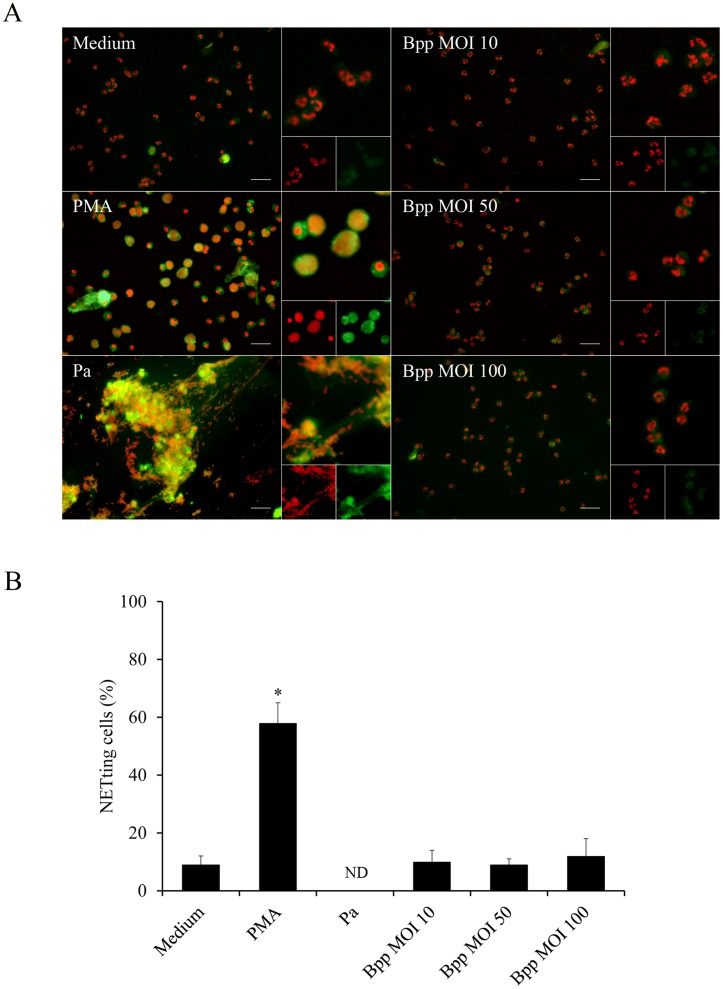
*B*. *parapertussis* precludes NETs release in resting neutrophils. Neutrophils were infected with non-opsonized *B*. *parapertussis* (Bpp) (MOI: 10, 50 or 100) or *P*. *aeruginosa* (Pa) (MOI: 10) for 4 h at 37°C. Samples were fixed and permeabilized prior to labeling the NE in green and neutrophil DNA with a red fluorescent dye. Neutrophils incubated with PMA or medium alone were used as a positive and negative control, respectively. Samples were analyzed by fluorescence microscopy and the number of NETting cells was determined by the ImageJ softaware. At least 500 cells were counted per sample. (A) Representative microscopy images of neutrophils 4 h post infection are shown. Scale bar: 20 μm. (B) The bars represent the percentage of neutrophils that underwent NETosis. The data represent the mean ± SD of three experiments with neutrophils from different donors. ND: non-determinable (NETotic cells are no longer observed; only big clusters of NETs are seen). The percentage of NETting cells in PMA treated neutrophils was significantly different from the percentage of NETting cells observed in the other samples (*P <0.05).

We next investigated whether the interaction of *B*. *parapertussis* with neutrophils induces NETosis, the other neutrophil extracellular bactericidal mechanisms. NETs induction was evaluated by fluorescence microscopy using a DNA intercalating dye and specific antibodies against NET-associated proteins. Fig 3 (S3 Data) shows that neutrophils that were incubated with medium alone did not undergo NETosis and exhibit a lobulated nucleus, NE is not colocalizing with the cell DNA, and no extracellular DNA structures can be observed. In contrast, neutrophils that underwent NETosis by PMA treatment or exposure to *P*. *aeruginosa* at an MOI of 10 showed a decondensed nucleus in which the chromatin colocalizes with NE and extracellular structures in which chromatin and NE are colocalizing, respectively. In contrast, *B*. *parapertussis* failed to induce NETosis at any MOI assayed, indicating that *B*. *parapertussis* does not induce NETosis in resting neutrophils.

**Fig 4:**
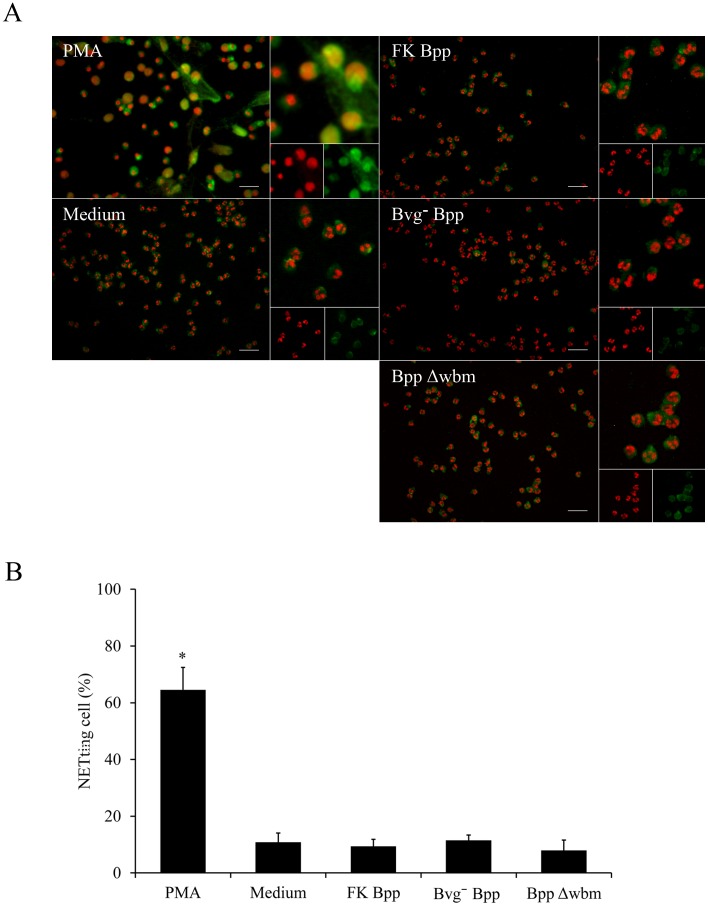
Preclusion of NETs release by *B*. *parapertussis* does not depend on Bvg-regulated factors, bacterial viability, or O antigen expression. Neutrophils were incubated with formalin inactivated *B*. *parapertussis* (FK Bpp), avirulent *B*. *parapertussis* (Bvg^-^ Bpp), or *B*. *parapertussis* Δwbm (BppΔwbm) (MOI: 100) for 4 h at 37°C. Neutrophils incubated with either medium alone or PMA were used as negative and positive controls, respectively. After incubation, samples were fixed and permeabilized prior to labeling the NE in green and neutrophil DNA with a red fluorescent dye. Samples were analyzed by fluorescence microscopy and the number of NETting cells was determined by the ImageJ softaware. At least 500 cells were counted per sample. (A) Representative microscopy images of each condition are shown. Scale bar: 20 μm. (B) The bars represent the percentage of neutrophils that underwent NETosis. The data represent the mean ± SD of three experiments with neutrophils from different donors. The percentage of NETting cells in PMA treated samples was significantly different from the percentage of NETting cells observed in the other samples (*P <0.05).

To evaluate whether the inhibitory effect of *B*. *parapertussis* on NETs formation was dependent on any of the Bvg-regulated virulence factors or bacterial viability, neutrophils were incubated with avirulent *B*. *parapertussis* or inactivated bacteria, respectively. As can been seen in Fig 4 (S4 Data), neither avirulent nor inactivated *B*. *parapertussis* induced NETosis. These results show that the lack of induction of NETs release in resting neutrophils by *B*. *parapertussis* is not dependent on a Bvg-regulated virulence factor or bacterial viability.

Since the O antigen has been found responsible for preclusion of neutrophil activation during the interaction with *B*. *parapertussis* [7], we investigated the relationship between the lack of NETs induction in resting neutrophils infected with *B*. *parapertussis* and the expression of the O antigen. As can been seen in Fig 4, neutrophils that were infected with an O antigen deficient mutant strain did not induce NETs formation, indicating that the O antigen is not involved in the preclusion of NET release.

**Fig 5:**
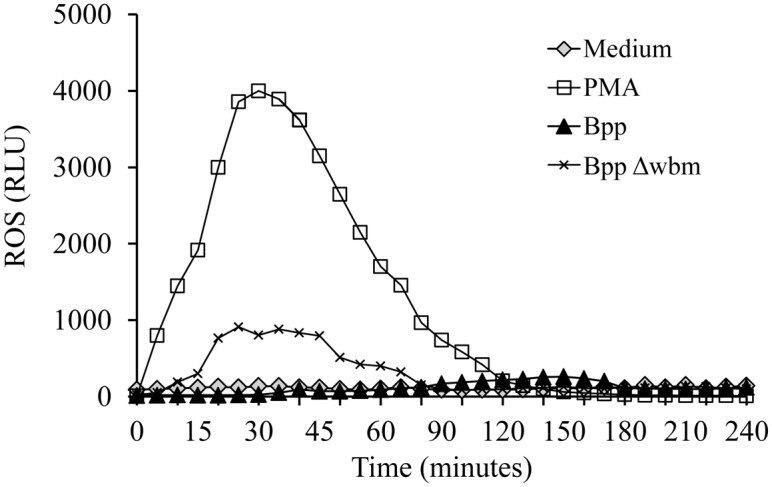
*B*. *parapertussis* does not induce ROS in resting neutrophils. *B*. *parapertussis* (Bpp) or *B*. *parapertussis* Δwbm (BppΔwbm) were incubated with neutrophils (MOI: 100) and luminol at 37°C. Neutrophils incubated with medium alone and luminol served as a negative control. Neutrophils incubated with PMA were used as positive controls. Chemiluminescence response was measured every 5 min. The data are representative of three independent experiments. RLU, relative light units.

The absence of NETosis upon neutrophil infection with the wild type *B*. *parapertussis* is in agreement with the lack of oxidative burst response observed in previous studies [7] and confirmed here for the whole period of time of the experiment (Fig 5). Since incubation of neutrophils with PMA is known to induce NETs formation in a ROS-dependent way [26], PMA-treated neutrophils were used as positive control. The ROS production was evaluated during 4 h after infection. Fig 5 (S5 Data) shows that PMA treatment induced an effective ROS production in neutrophils while *B*. *parapertussis* or medium alone did not. The O antigen defective mutant of *B*. *parapertussis*, however, induced a low but significant level of oxidative burst response (Fig 5, S5 Data) but did not promote NETs release. These results might indicate that ROS induction or, at least, this level of ROS, is not sufficient to induce NETosis.

### *B*. *parapertussis* inhibits NETs release in PMA stimulated neutrophils

**Fig 6:**
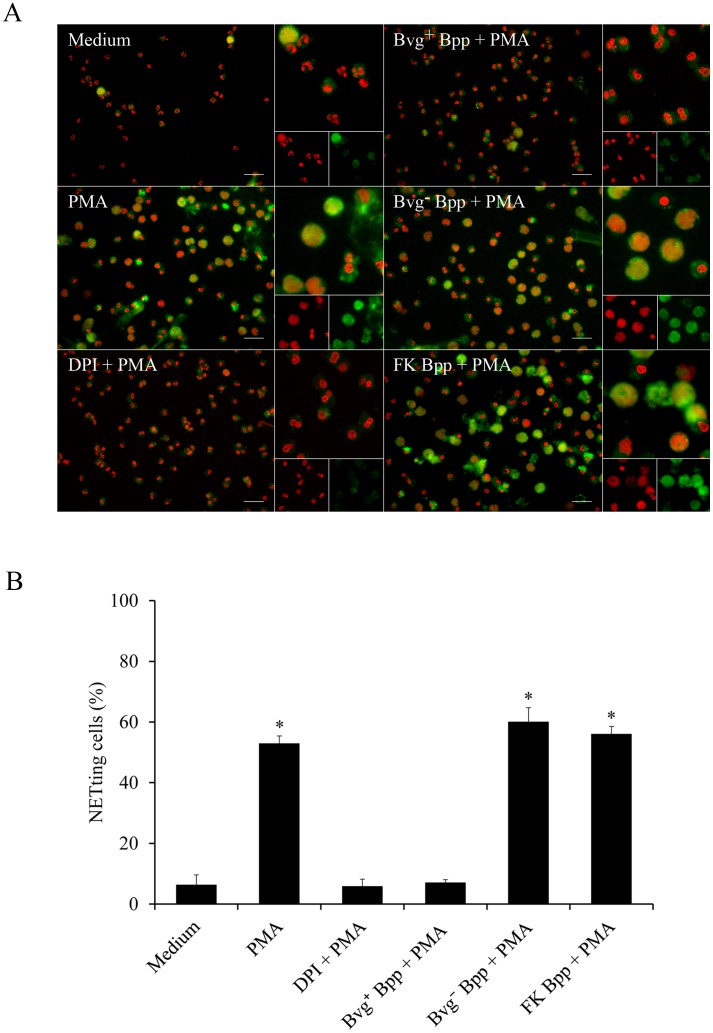
*B*. *parapertussis* inhibits PMA-induced NETs through a Bvg-regulated factor. Neutrophils were incubated with virulent *B*. *parapertussis* (Bvg^+^ Bpp), formalin inactivated *B*. *parapertussis* (FK Bpp), avirulent *B*. *parapertussis* (Bvg^-^ Bpp) (MOI 10), or DPI for 30 min at 37°C prior to PMA treatment. Neutrophils incubated with medium alone were used as negative control. Neutrophils incubated with PMA alone were used as positive control. Neutrophils treated with DPI 30 min before PMA treatment were used as control of PMA-induced NETs inhibition. Four hours post infection samples were fixed and permeabilized prior to labeling the NE in green and neutrophil DNA in red. Samples were analyzed by fluorescence microscopy and the number of NETting cells was determined by the ImageJ software. At least 500 cells were counted per sample. (A) Representative microscopy images of each condition are shown. Scale bar: 20 μm. (B) The bars represent the percentage of neutrophils that underwent NETosis. The data represent the mean ± SD of three experiments with neutrophils from different donors. * indicates a P value <0.05 for comparison to results for neutrophils incubated with medium alone.

**Fig 7:**
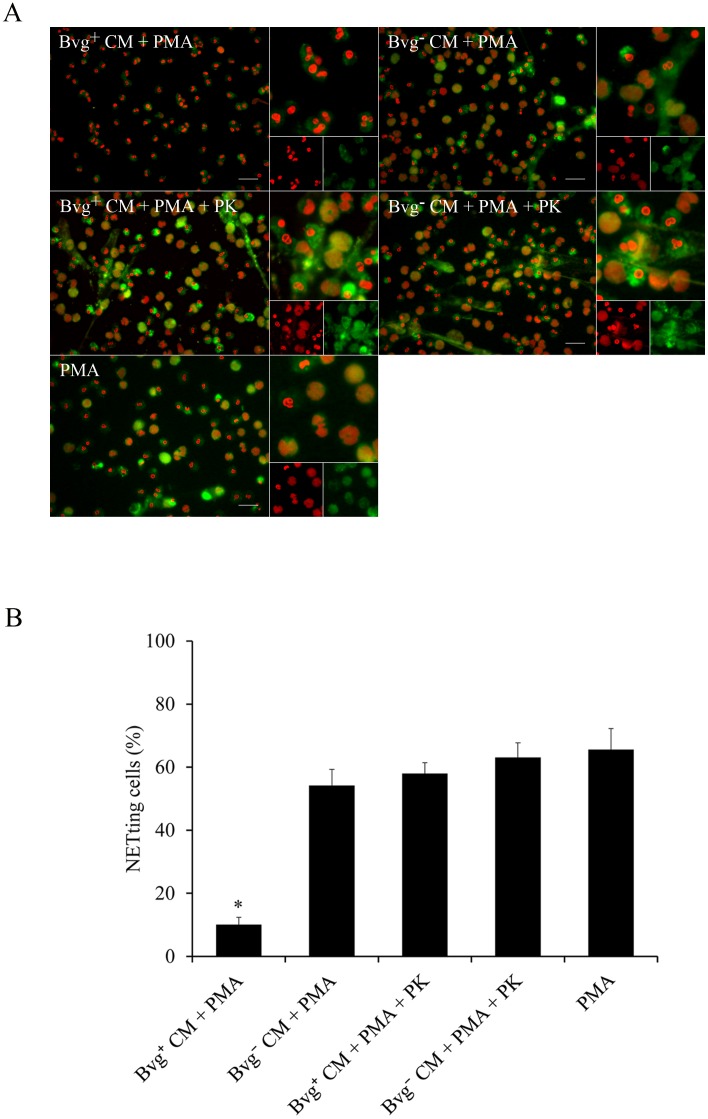
A *B*. *parapertussis* secreted Bvg-regulated virulence factor is involved in PMA-induced NETs inhibition. CM was prepared by incubating 2x10^8^ CFU/ml of virulent *B*. *parapertussis* (Bvg^+^ CM) or avirulent *B*. *parapertussis* (Bvg^-^ CM) in medium for 2 h. In some samples CM was treated with proteinase K (PK) (100 μg/mL) for 60 min at 37°C prior use. Neutrophils were incubated with each CM 30 min before treatment with PMA. Neutrophils incubated with PMA alone were used as positive control. Four hours after PMA treatment samples were fixed and permeabilized prior to labeling the NE in green and neutrophil DNA in red. Samples were analyzed by fluorescence microscopy and the number of NETting cells was determined by the ImageJ softaware. At least 500 cells were counted per sample. (A) Representative microscopy images of each condition are shown. Scale bar: 20 μm. (B) The bars represent the percentage of neutrophils that underwent NETosis. The data represent the mean ± SD of three experiments with neutrophils from different donors. The percentage of NETting cells in neutrophils incubated with Bvg^+^ CM prior to PMA treatment was significantly different from the percentage of NETting cells observed in the other samples (*P <0.05).

NETosis is induced *in vivo* by a wide range of stimuli that includes different cytokines, bacterial components or a combination of these and other signals [21]. To examine whether *B*. *parapertussis* is able to actively inhibit NETs formation neutrophils infected with *B*. *parapertussis* (MOI: 10) for 30 min were treated with PMA and 4 h later NETs formation was evaluated by fluorescence microscopy. Neutrophils treated with or without DPI for 30 min before PMA treatment were used as a control of PMA-induced NETs inhibition and positive control, respectively. Fig 6 (S6 Data) shows that *B*. *parapertussis* efficiently inhibits PMA-induced NETs. In order to determine whether this inhibition depends on bacterial virulence factors or bacterial viability we investigated the effect of avirulent *B*. *parapertussis* or inactivated bacteria on PMA induction of NETs. As can be seen in Fig 6 (S6 Data), PMA treatment induced NETosis in neutrophils previously incubated with either inactivated or avirulent *B*. *parapertussis*, suggesting that the active form of a Bvg-regulated factor is involved in the observed inhibition. To further elucidate whether the inhibitory factor was released into the surrounding medium, a CM was prepared by incubating virulent bacteria (Bvg^+^ CM) or avirulent bacteria (Bvg¯ CM) in the medium for 2 h. Each CM was then filtered to remove the bacteria and further used to treat the neutrophils 30 min before PMA addition. Fig 7 shows that treatment of neutrophils with Bvg^+^ CM prior to PMA stimulation inhibited PMA induction of NETs. On the other hand, incubation of neutrophils with the Bvg¯ CM did not inhibit PMA-induced NETs release (Fig 7, S7 Data). Finally, both Bvg^+^ CM and Bvg¯ CM were treated with proteinase K prior to incubation with neutrophils and further treated with PMA. Proteinase K treatment abolished the capacity of the Bvg^+^ CM to inhibit NETs formation in response to PMA, but had no effect on Bvgˉ CM (Fig 7, S7 Data), indicating that the factor blocking NET formation is a protein sensitive to proteinase K.

**Fig 8:**
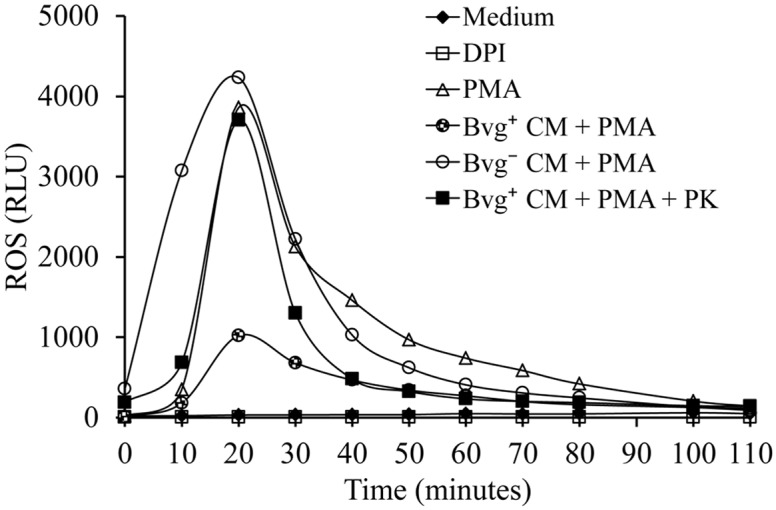
A Bvg-regulated virulence factor secreted into extracellular medium is involved in the preclusion of ROS generation in PMA treated neutrophils. CM prepared by incubating 2x10^8^ CFU/ml of virulent *B*. *parapertussis* (Bvg^+^ CM) or avirulent *B*. *parapertussis* (Bvg^-^ CM) in medium for 2 h were used to incubate neutrophils 30 min before treatment with PMA. In some cases the CM was treated with proteinase K (PK) (100 μg/mL) for 60 min at 37°C, and further used to incubate neutrophils 30 min before treatment with PMA. Neutrophils incubated with medium alone and luminol served as a negative control. Neutrophils treated with DPI 30 min before PMA treatment were used as a control of PMA-induced NETs inhibition (DPI). Neutrophils incubated with PMA alone were used as positive control. Chemiluminescence responses were measured every 10 min in the presence of luminol. Data are representative of three independent experiments. RLU, relative light units.

Having in mind that PMA induces NOX/ROS-dependent NETs formation [26], we next evaluated whether Bvg^+^ CM inhibits ROS production in PMA treated neutrophils. To this end, neutrophils were treated with either Bvg^+^ CM or Bvg^-^ CM for 30 min prior to PMA addition. Generation of ROS was measured for at least 2 h post treatment. Neutrophils stimulated with PMA were used as a positive control for ROS production. Neutrophils incubated with the NOX inhibitor DPI prior to PMA addition were used as a control for inhibition of PMA-induced ROS. The results presented in Fig 8 (S8 Data) showed that Bvg^+^ CM inhibited PMA induced ROS in neutrophil, whereas the Bvg^-^ CM did not. Again, proteinase K treatment of Bvg^+^ CM abrogated the inhibition of PMA-induced ROS. Altogether these results suggest that Bvg-regulated proteins released into the extracellular medium prevent PMA induction of NETs release by NOX/ROS inhibition.

### *B*. *parapertussis* CyaA inhibits ROS-dependent NETs generation

**Fig 9:**
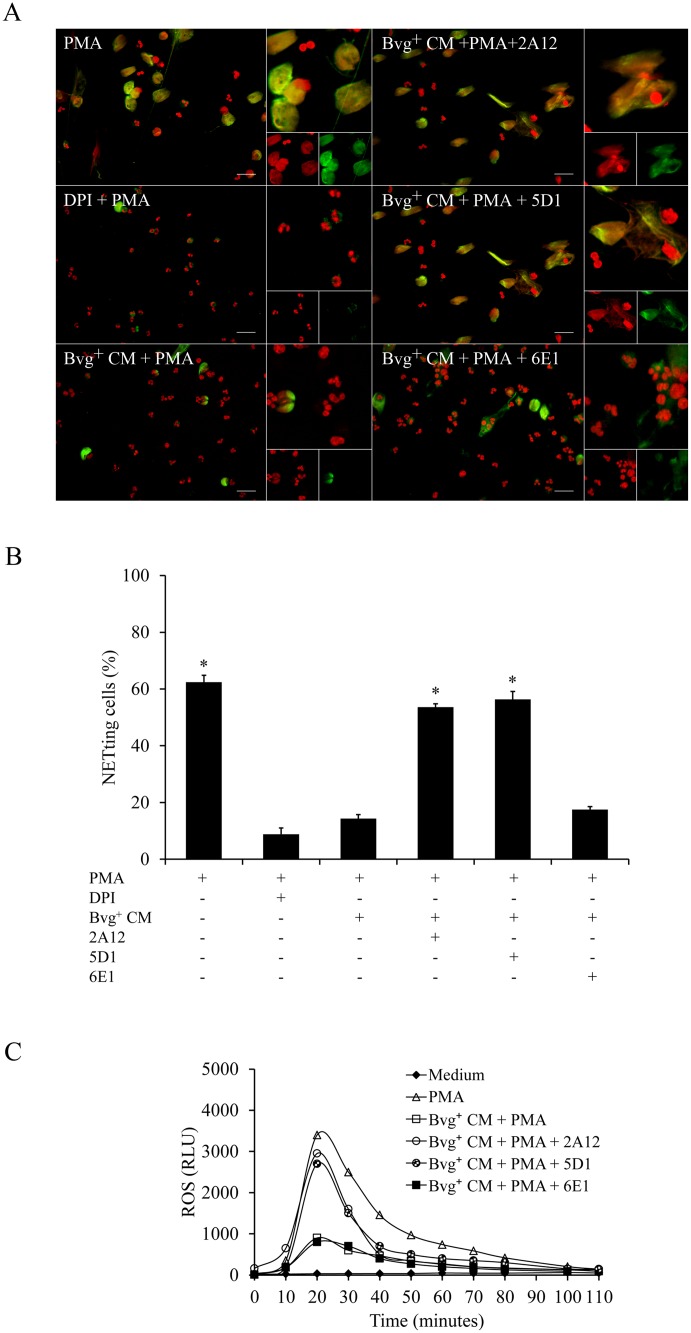
*B*. *parapertussis* adenylate cyclase inhibits NETs and ROS generation in PMA treated neutrophils. CM prepared by incubating 2x10^8^ CFU/ml of virulent *B*. *parapertussis* (Bvg^+^ CM) in medium for 2 h was used to incubate neutrophils 30 min before treatment with PMA. In some cases CM was treated with antibodies against both activities of adenylate cyclase toxin (2A12), or antibodies against either adenylate cyclase (5D1) or the haemolytic (6E1) activity. Four hours post incubation samples were fixed and permeabilized prior to labeling the NE in green and neutrophil DNA in red. Neutrophils incubated with DPI 30 min before PMA treatment were used as a control of inhibition of PMA-induced NETs (DPI). Neutrophils incubated with PMA alone were used as a positive control. The percentage NETing cell was determined using the ImageJ softaware. (A) Representative microscopy images of each condition are shown. Scale bar: 20 μm. (B) The bars represent the percentage of neutrophils that underwent NETosis. The data represent the mean ± SD of three experiments with neutrophils from different donors. * indicates a P value <0.05 for comparison to results for control of PMA-induced NETs inhibition (DPI). (C) Chemiluminescence responses of neutrophils incubated with 2A12, 5D1, or 6E1-treated Bvg^+^ CM prior to the incubation with PMA. Neutrophils incubated with medium alone and luminol served as a negative control. Neutrophils incubated with PMA alone were used as positive control. Chemiluminescence was measured every 10 min during the whole time of the experiment. Data are representative of three independent experiments. RLU, relative light units.

Previous studies have shown that *B*. *pertussis* CyaA inhibits PMA-generated NETs by ROS inhibition [45]. *B*. *parapertussis* also expresses CyaA in a Bvg-regulated manner. This protein toxin is released into the extracellular medium and could be responsible for the observed inhibition of PMA stimulated NETs by Bvg^+^ CM. To investigate the role of CyaA, the Bvg^+^ CM was incubated with a monoclonal antibody against CyaA (2A12), which partially blocks both the adenylate cyclase and hemolytic activities of this toxin. Neutrophils were incubated with this 2A12-treated Bvg^+^ CM 30 min before PMA treatment and NETs formation was determined by fluorescence microscopy as described above. As can be seen in Fig 9A and 9B, the 2A12 antibody abrogated the inhibitory effect of the Bvg^+^ CM on PMA-induced NETs. To evaluate which of the toxin activities is responsible for this inhibition, Bvg^+^ CM was incubated with either a monoclonal antibody against adenylate cyclase activity (5D1), which prevents translocation of the enzyme catalytic domain into the cell without affecting the binding of the toxin to cells, or with a monoclonal antibody against the hemolytic activity of this toxin (6E1), which binds to the hemolytic domain of the enzyme without impairing intracellular toxin-induced cAMP accumulation. Fig 9A and 9B (S9 Data) shows that 2A12 and 5D1 antibody abolished PMA-induced NETs inhibitory activity of Bvg^+^ CM. On the other hand, treatment with 6E1 had no effect on the inhibitory activity of Bvg^+^ CM. These results indicate that the inhibition of PMA-induced NETs by *B*. *parapertussis* depends on adenylate cyclase activity of CyaA. In agreement with these results, incubation with 2A12 or 5D1 suppressed the inhibitory activity of Bvg^+^ CM on the PMA-induced ROS (Fig 9C, S10 Data), while incubation with 6E1 had no effect. These results demonstrate that CyaA, through its adenylate cyclase activity, prevents PMA induction of NETs release through NOX/ROS inhibition.

### NETs trap and kill *B*. *parapertussis*

**Fig 10:**
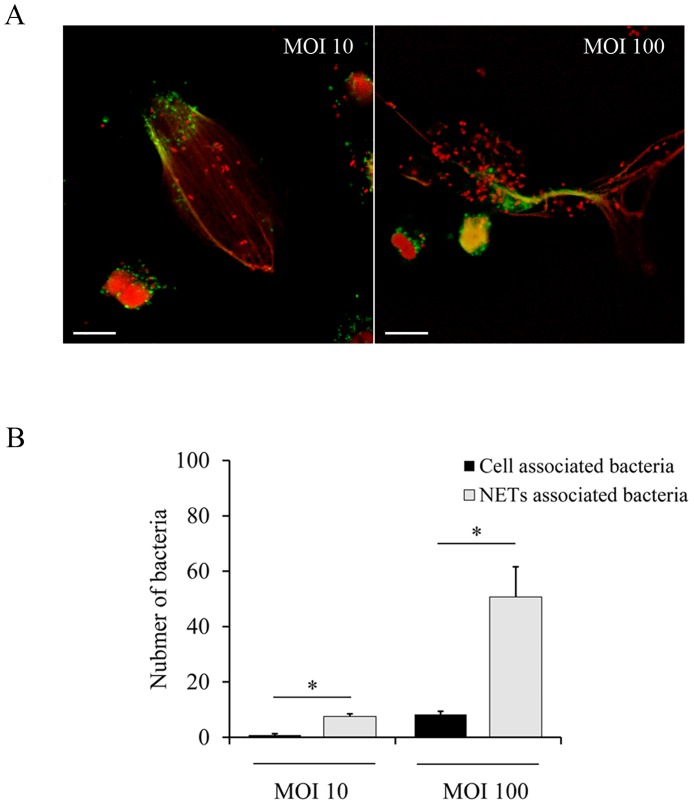
NETs can trap and immobilize *B*. *parapertussis*. Neutrophils were incubated with PMA (100 nM) for 1 h prior to infection with *B*. *parapertussis* (MOI 10 or 100) for 4 h at 37°C. Next, neutrophils were fixed and permeabilized prior to labeling the NE in green and both the neutrophil and bacterial DNA in red. (A) Representative confocal microscopy images of three independent experiments are shown. Scale bar: 10 μm. (B) The number of associated bacteria to both NETs- and non-NETting neutrophils was determined by analyzing at least 20 fields per sample. The bars represent the number of associated bacteria to either NETs- or non-NETting neutrophils. The data represent the mean ± SD of at least three experiments with neutrophils from different donors. The number of associated bacteria to NETs was significantly different to the number of associated bacteria to non-NETting cells at both MOI assayed (*P <0.05).

Although *B*. *parapertussis per se* does not seem to induce NETs, NETs can be induced by many different stimuli *in vivo* at the site of infection [21]. We investigated whether these extracellular structures can trap and kill *B*. *parapertussis*. To this end, NETs were induced by PMA (100 nM) treatment before adding *B*. *parapertussis*. The capacity of NETs to immobilize *B*. *parapertussis* was examined by confocal microscopy. As can been seen in Fig 10 (S11 Data), NET structures stained for DNA (red) and NE (green) are surrounding *B*. *parapertussis* bacteria (red; stained for DNA), indicating that NETs can trap *B*. *parapertussis*. Fig 10 shows the number of bacteria attached to NETs as compared with the number of bacteria taken up by non-NETting neutrophils at two different multiplicities of infection, 10 and 100. In both cases NETs captured more than 80% of the total number of bacteria (90.07% ± 11.37 and 85.96% ± 18.46, respectively).

**Fig 11:**
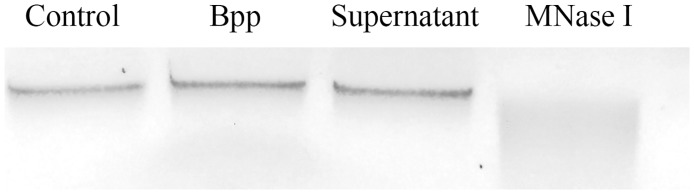
*B*. *parapertussis* does not have DNAse activity. Gel electrophoresis analysis of DNA (300 ng) incubated 1 h a 37°C with PBS (PBS), *B*. *parapertussis* (Bpp), culture supernatant of *B*. *parapertussis* (supernatant), and MNase I (500 mU/ml). Representative images are shown.

Several pathogens may escape from NETs by breaking these structures by a self-DNase activity [46–48]. We investigated whether *B*. *parapertussis* can breakdown extracellular DNA by determining bacterial DNase activity both in the whole cell bacteria and in bacterial-free culture supernatant by incubation with purified neutrophil DNA for 60 min. DNA integrity was evaluated by agarose gel electrophoresis. As can be seen in the Fig 11 we found neither *B*. *parapertussis* cell-associated nor secreted DNAase activity, suggesting that this bacterium does not have this mechanism for NETs evasion.

**Fig 12:**
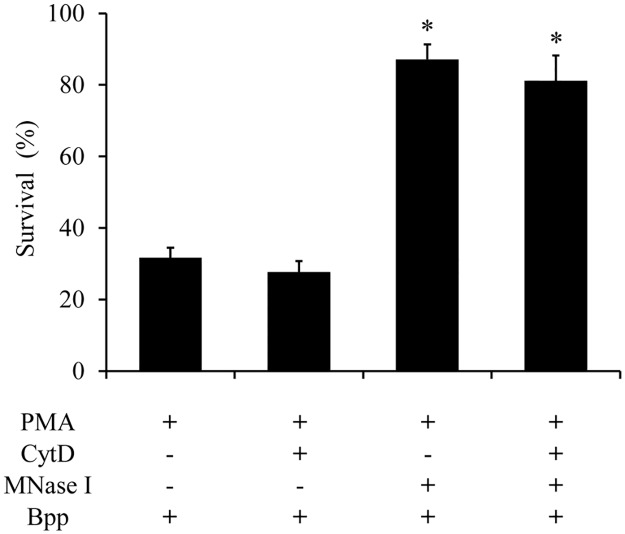
*B*. *parapertussis* is killed by NETs. Neutrophils were incubated with PMA (100 nM) for 1 h at 37°C to induce NETs release. Next, neutrophils were treated with cytochalasinD (100 μg/ml; CytD), MNase I (500 mU/ml), or a combination of both for 30 min at 37°C. Neutrophils were then incubated with *B*. *parapertussis* (Bpp) (MOI 100) for 3 h at 37°C. Next, all samples were treated with MNase for 20 min to release entrapped bacteria from NETs and serial dilutions of cell lysates were rapidly plated onto bBGA to enumerate CFU. The percentage of viable bacteria was referred to bacterial inoculums. The number of viable *B*. *parapertussis* in samples treated with MNase I prior bacterial infection was significantly different from that found in samples that were not treated with MNase I. * indicates a P value <0.05 for comparison to results for PMA-treated neutrophils infected with Bpp.

Since some microorganisms can be trapped but not killed by NETs [20], we investigated *B*. *parapertussis* survival after NETs encounter. Again, NETs were induced by PMA treatment before infection with *B*. *parapertussis*. Bacterial survival was evaluated 3 h after infection. CFU counts of bacterial inoculum was determined and set as 100%. As can be seen in Fig 12 (S12 Data), PMA-induced NETs significantly reduced the number of viable bacteria. In order to discriminate between bacteria killed by NETs or phagocytosis, neutrophils were either incubated with CytD or MNase I, prior to incubation with bacteria. CytD treatment inhibits phagocytosis, while MNase I treatment breaks down NETs structure. A combination of MNase I and CytD was also used to exclude both killing effects. Fig 12 show that the reduction in the number of viable *B*. *parapertussis* in PMA treated neutrophils was due to the bactericidal activity of NETs.

## Discussion

Neutrophils are rapidly recruited by microbial infection, and have a variety of anti-bacterial mechanisms. To survive this onslaught, many microbial pathogens have evolved strategies to evade or counteract these mechanisms to successfully infect the host. We have previously found that in the absence of opsonic antibodies *B*. *parapertussis* avoids neutrophil intracellular bactericidal mechanism by multiple mechanisms [7]. According to those results, the great majority of bacteria remain extracellularly, where they might be subject to neutrophil degranulation and NETs release. In this study we investigated the relevance of these neutrophil extracellular bactericidal mechanisms in the control of *B*. *parapertussis*.

Degranulation usually takes place once phagocytosis is initiated and neutrophil cytoplasmic granules are fused with nascent unclosed phagosomes leading to phagolysosome formation [11, 12]. This process eventually leads to the release of bactericidal granular components into the surrounding medium ultimately killing extracellular bacteria. The results of this study showed that at a multiplicity of infection as high as 300 bacteria per neutrophil there was not significant degranulation, as determined by the lack of β-glucuronidase activity in the cell-free supernatants of infected cells. A mutant lacking O antigen stimulated degranulation, indicating both that *B*. *parapertussis* can stimulate this neutrophil response and that the O antigen molecule blocks it. We had previously shown that the O antigen targets the bacteria to the lipid raft domains on the host cell membrane [7]. Bacterial entry through lipid raft platforms is involved in the evasion of intracellular signaling pathways linked to the activation of neutrophil bactericidal mechanisms [49, 50]. Soon after bacteria-neutrophil contact confocal studies showed the wild type strain of *B*. *parapertussis* in close contact with lipid rafts at the plasma membrane of neutrophils, and the absence of specific and azurophilic granules in the bacterial surroundings at the site of entry during phagocytosis. Conversely, the *B*. *parapertussis* O antigen-deficient mutant that did not colocalize with lipid rafts domains was found surrounded by a great number of specific and azurophilic granules. The fusion of these cytoplasmic granules with unclosed phagosomes containing *B*. *parapertussis* O antigen mutant probably contributed to the observed robust neutrophil degranulation. This key role of the O antigen in the inhibition of neutrophils degranulation adds to the already long list of the bacterial protective characteristics of this molecule.

IgG opsonization of *B*. *parapertussis* overcomes O antigen-mediated inhibition of degranulation, as determined by the high release of β-glucuronidase in the cell supernatant of neutrophils infected with IgG opsonized *B*. *parapertussis*. This result is in agreement with the finding that IgG-opsonized bacteria do not enter through lipid rafts domains, are trafficked to lysosomes, and strongly activate neutrophils [7]. These results stress the need for opsonic antibodies at the site of infection not only to promote efficient phagocytosis and intracellular bacterial inactivation [7] but also to induce extracellular bacterial killing through neutrophil degranulation.

NETs represent a complementary approach to contain and kill extracellular bacteria. Several stimuli can activate specific signaling pathways leading to NETotic cell death and the release of a mixture of granular content associated with DNA fibers in the extracellular medium [9]. This is a potent antimicrobial mechanism since the NETs immobilize and eventually kill microbes that are in the surrounding of the neutrophil. Bacterial components are strong inductors of NETs [51]. However, we observed that *B*. *parapertussis* interactions with resting neutrophils do not induce NETs production. According to our results *B*. *parapertussis* does not induce NETosis even at high multiplicities of infections at which fungi, virus, protozoa, and several bacterial pathogens induce a strong NETosis [21]. Some pathogens use active mechanisms to circumvent this neutrophil extracellular killing mechanism, like the secretion of nucleases that break down NETs [46–48]. But *B*. *parapertussis* did not express detectable cell-associated nor secreted DNase activity. It does expresses a potent set of Bvg-regulated virulence factors that mediates several immune evasion mechanisms, but none of these virulence factors were found implicated in the lack of NETs induction in resting neutrophils. The lack of induction of NETs in resting neutrophils by *B*. *parapertussis* was not even dependent on the viability of the bacteria as was proved by infection with inactivated *B*. *parapertussis*.

Bacterial LPS is a strong NETs inductor [28, 52]. However, bacterial pathogens have coevolved with their host to hide themselves from recognition by the immune system. In some pathogens adaptation to humans has provided selective pressure to modify surface structures like LPS. Previous studies have shown that *B*. *parapertussis* LPS has specific modifications that determine its low stimulatory activity of the immune system [7, 53, 54]. Particularly, the LPS O antigen decoration has been found responsible for the prevention of neutrophil oxidative burst by *B*. *parapertussis* [7]. Since ROS production is required by almost all stimuli that lead to NETs release [25, 26], we hypothesized that the O antigen was involved in the lack of NETs induction. However, the O antigen deficient *B*. *parapertussis*, although able to induce a small but significant oxidative burst, did not promote NETs release. These results seem to indicate that either ROS stimulation induced by the O antigen mutant strain is not strong enough to promote NETs release (like observed for *B*. *pertussis* [45]) or other required mechanisms are not triggered during this bacterial-cell interaction, i.e. autophagy activity [26]. In summary, the lack of NETs induction by *B*. *parapertussis* seems associated to the low stimulatory potential of its LPS as previously found for other pathogens like *Brucella abortus* [55].

Neutrophil activation and NETs release can be initiated by several stimuli such as a combination of cytokines, or bacterial components (LPS) plus proinflamatory cytokines [21], among others. In this study, we challenged the ability of *B*. *parapertussis* to inhibit NETs release in the presence of the most potent NETs inducer, PMA [26]. We observed that *B*. *parapertussis* is able to inhibit PMA-induced NETosis by preventing PMA-induced oxidative burst. This inhibition proved dependent on the CyaA released into the extracellular medium indicating that NETs inhibition does not require the close proximity of bacteria with neutrophils. CyaA has two different biological activities, a hemolysin activity implicated in cell lysis, and the adenylate cyclase activity that intoxicates target cell by increasing the intracellular level of cAMP. By using specific monoclonal antibodies against the different CyaA activities, we found that the adenylate cyclase activity is responsible for both ROS and NETosis inhibition in PMA-stimulated neutrophils. Our findings are in agreements with those of Eby, *et al*. who demonstrated that CyaA inhibits PMA-induced NOX activity [45]. Inhibition of NOX prevents ROS generation and chromatin decondensation which is essential for NET formation [26]. In this previous work, Eby, *et al*. [45] suggested that CyaA inhibits NOX activity through the inhibition of the Raf-MEK-ERK signaling pathway, which is involved in the assembly and activation of the NOX. Accordingly, we observed that CyaA arrests PMA treated neutrophils in a delobulated nuclei stage (Fig 6), which is typical in the inhibition of Raf-MEK-ERK signaling pathway [56]. Therefore, like *B*. *pertussis*, *B*. *parapertussis* inhibition of PMA-induced NETs probably depends on an active inhibition of Raf-MEK-ERK signaling pathway mediated by CyaA.

While many human pathogens have been shown to induce NETs formation, and some have mechanisms to escape from NETs, *B*. *parapertussis* is the first reported human pathogen that does not induce NETs and had the ability to inhibit NET formation. Through two different mechanisms, one related to the lack of proper NET-inducer stimuli and the other related to a virulence factor-mediated inhibition, *B*. *parapertussis* control an essential neutrophil mechanism against microbial infections. This dual strategy to avoid NETs induction has only been described in the Gram-positive bacterium *Lactobacillus rhamnosus* [57], but to our knowledge there is no precedent in Gram-negative bacterium. Interestingly, unlike other pathogens that avoid neutrophil extracellular killing by escaping from NETs, the capacity of *B*. *parapertussis* to block NETs induction can prevent an exacerbated inflammatory response originated by the release of tissue-damaging molecules after neutrophil lysis during NETs release. Furthermore, NETs inhibition mediated by an excreted virulence factor might be useful for *B*. *parapertussis* at the site of infection where several potential soluble NETs inducer stimulus may be present. By being able to control NETs release without intimate contact with the cell *B*. *parapertussis* generates a relatively large environment free of NETs. This NETs release control might be quite relevant for *B*. *parapertussis* survival since NETs can efficiently trap and kill this bacterium. On the other hand, the inhibition of neutrophil death mechanisms like NETosis, might be advantageous for a bacterium like *B*. *parapertussis* that is able to remain alive inside the cell as a transient intracellular stage.

We have previously shown that opsonic antibodies are required for neutrophils and macrophages to efficiently phagocytose and kill *B*. *parapertussis* [6, 7]. The results obtained in this study demonstrate that without opsonizing antibodies this pathogen might also avoid extracellular bactericidal mechanisms of neutrophils. These results are consistent with previous studies performed in a murine model of infection that demonstrated that *B*. *parapertussis* clearance requires both the presence of neutrophils and specific antibodies against this bacterium [58]. The lack of either precludes bacterial clearance.

Adenylate cyclase has been proposed as a pertussis vaccine candidate. In this work, we showed that antibodies against *B*. *pertussis* adenylate cylcase toxin neutralize *B*. *parapertussis* CyaA released into the extracellular medium allowing NETs induction by stimulated neutrophils. Therefore, in the absence of proper opsonic antibodies to induce a strong neutrophil bactericidal activity, antibodies against this toxin might be valuable for the control of *B*. *parapertussis*.

## Supporting Information

S1 Data Raw data of Fig 1. (XLSX)

S2 Data Raw data of Fig 2. (XLSX)

S3 Data Raw data of Fig 3. (XLSX)

S4 Data Raw data of Fig 4. (XLSX)

S5 Data Raw data of Fig 5. (XLSX)

S6 Data Raw data of Fig 6. (XLSX)

S7 Data Raw data of Fig 7. (XLSX)

S8 Data Raw data of Fig 8. (XLSX)

S9 Data Raw data of Fig 9B. (XLSX)

S10 Data Raw data of Fig 9C. (XLSX)

S11 Data Raw data of Fig 10. (XLSX)

S12 Data Raw data of Fig 12. (XLSX)
